# Assisting Strategies of the Parents of Students with Special Educational Needs in the Emergency Remote Learning in Poland

**DOI:** 10.3390/ijerph19148783

**Published:** 2022-07-19

**Authors:** Tomasz Knopik, Anna Błaszczak, Urszula Oszwa, Renata Maksymiuk

**Affiliations:** 1 Institute of Psychology, Maria Curie-Sklodowska University, 20-400 Lublin, Poland; anna.blaszczak@umcs.pl; 2 Institute of Pedagogy, Maria Curie-Sklodowska University, 20-400 Lublin, Poland; urszula.oszwa@umcs.pl; 3 Institute of Psychology, Jagiellonian University, 31-007 Kraków, Poland; renata_maksymiuk@o2.pl

**Keywords:** remote learning, parental involvement, special educational needs, COVID-19 pandemic

## Abstract

The study had four objectives: (a) identifying and characterizing strategies for involving parents of students with SEN (students with special educational needs) in remote education during the COVID-19 pandemic; (b) comparing these strategies with those used by parents of students without SEN (non-SEN students); (c) identifying predictors of parental involvement in the remote education of students with SEN; (d) checking whether the identified strategies differentiate the perceived barriers and benefits of remote learning. In total, 421 parents of primary school students participated in the study, 83 of whom (20%) were parents of children with SEN (SEN group). Based on the factor analysis of the results (respondents completed a 66-item electronic questionnaire), three main strategies for parental involvement in children’s remote education were identified: (1) committed teacher (CT), with 40% in the SEN group and 55% in the non-SEN group; (2) autonomy-supporting coach (ASC), with 22% in the SEN group and 26% in the non-SEN group; (3) committed teacher and reliever (CTR), with 38% in the SEN group and 19% in the non-SEN group. The strongest predictor of parental involvement with SEN students in the role of committed teacher was excessive demands from school. Parents whose children showed low motivation to learn were the most likely to do some of their children’s school tasks for them and apply the committed teacher and reliever strategy. The positive aspects of remote education were mostly noticed by moderately committed parents who gave their children a lot of autonomy (autonomy-supporting coaches). The obtained results can be included in the optimization of schools’ activities in terms of organizing remote education for students with SEN and cooperating with parents.

## 1. Introduction

The time of the COVID-19 pandemic was known as the period of emergency remote learning in many countries. Most governments restricted the functioning of educational institutions and transferred the educational system to virtual networks and homes. In most cases, a significant increase in the time devoted by parents to participation in their children’s education was expected [1] due to the need to take over the role of teachers or therapists. However, parents might take part in the remote teaching of their own children by applying various strategies which could have a significant impact on their knowledge and skills.

### 1.1. Parental Involvement in Children’s Education

Parental involvement (PI) in the development of children can be understood as activities aimed at supporting their emotional, social and academic experience [2]. It can have significant impact on students’ achievements and skills [3]. Hoover-Dempsey and Sandler [4] indicate different consequences of different types of PI methods. Some activities aim at immediate, direct effects. These are mainly used to explain instructions, clarify tasks, indicate examples of sample materials, help children master factual knowledge or improve incorrect answers. Some other actions aim to develop learning skills and shape attitudes that will foster lifelong learning.

Studies have revealed that PI is strongly linked to students’ academic success in the school system. It can lead to an increase in students’ success traits, such as punctuality and adequate behavior, awareness of the complexity of school relationships and the rules governing community functioning, higher self-regulation and adaptation to work and higher educational ambitions [5]. What is evident in meta-analyses is a significantly greater effect of indirect PI (educational aspirations, counselling, talking about school) on test-measured achievements than on direct involvement in school activity [6].

For SEN students, the intensity and scope of PI are certainly greater than for non-SEN students [3]. The category of special needs assumes additional, extraordinary support of the school and the parents. In relation to SEN students, PI might support them too much, thus limiting autonomy and initiative. Research conducted by Szumski and Karwowski [3] among 1500 students, including over 300 SEN students, showed that parents of SEN students more often used a mechanical strategy of engagement that was focused on ad hoc results in the form of positive school grades and performance of homework set by the teacher. Moreover, it was found that a higher level of such PI in assisting children in home schooling predicted lower school achievement for both SEN and non-SEN students.

### 1.2. The Specificity of Remote Learning and Its Main Challenges

Remote learning takes place outside of traditional schools and does not require the physical presence of the student and teacher in the same space [7]. According to Zawacki-Richter and Anderson [8], remote education requires global support systems and access to technology and theories that describe its specificity. On the micro level, remote education requires detailed curriculum development so that it is possible to implement the planned learning outcomes in virtual reality, to prepare and use materials adapted to remote lessons, to use technology efficiently and to ensure that teachers and students are well motivated.

Such features of remote education itself raise many challenges, but the suddenness and compulsion to use this form of education as an emergency that resulted from the COVID-19 pandemic aggravated the situation even more [9]. It is worth emphasizing that the paradigm of remote education has changed: from the existing blended learning model (in which remote learning was a supplement to traditional learning and was practiced only in a few institutions) to learning being conducted entirely remotely and as the rapid emergency. This caused communication and methodological chaos among school principals, teachers, students and their parents, while clarity of rules, plans, curricula and instructions should be the key to successful ERL [7]. Most of the teaching resources and instructions provided by teachers for ERL were not adapted to the specifics of this form of education; this was one of the most significant barriers to remote learning [10]. Teachers viewed the time-consuming preparation of remote learning resources during the COVID-19 pandemic as one of their main difficulties [11]; this primarily involved developing materials for the whole class without taking into account SEN students, without personalizing materials for students and without responding to their individual needs [12].

The next significant challenge for ERL is the attitude and motivation of students [13]. It has been assumed that ERL could be most often deliberately chosen by students for specific reasons (willingness to acquire specific knowledge or master skills, inability or reluctance to use traditional learning methods due to health or financial reasons, etc.). Therefore, Dron [13] described remote learners’ intrinsic motivations to learn, which is supposed to result from the greater autonomy they experience while learning remotely, namely choosing what, when and how they learn [14]. Another barrier to ERL frequently mentioned by students was social-deprivation [15]. Despite the technical possibilities provided by the applications they use, teachers did not offer their students tasks and group work that required cooperation [16], whilst normally, such active and engaging learning activities appeared to be more effective than learning individually with the use of materials prepared by teachers [15]. Remote learning undoubtedly presents challenges for students with SEN. Research conducted in the USA by Averett [17] among families of students with disabilities indicated that most of them struggled with two types of difficulties: (1) the children’s specific needs while learning at home were different from those at school (new, previously unknown problems emerged), and (2) schools did not provide comprehensive distance support. The transition to remote learning for students with disabilities introduced new sources of stress for both students and parents, and the high need for educational support was surprising and impossible for families to meet [18]. Some researchers have indicated that implementing remote education was particularly difficult for students with autism, who require a high level of routine and regularity [19]. A number of studies focusing on parents of children with SEN have found a lack of appropriate materials and resources tailored to the needs of these students and the need for the parent to step into the role of expert specialist, despite a lack of competence in this area [20,21,22].

### 1.3. Functioning of SEN Students in the Inclusive Model of Education

In the Polish education system, the group of SEN students has been precisely described by the catalogue of difficulties and disabilities included in 12 categories, such as, for example, a student with autism spectrum disorders, intellectual disability, learning difficulties and somatic diseases. The key to determining the possession of SEN is the student’s experience of barriers in making learning progress [23]. The identification of barriers starts with educational support provided by teachers, school psychologists, educators and speech therapists, as well as an external psych-pedagogue counseling center in the way adjusted to students’ needs. 

Inclusive education refers to a method of educating SEN children in a regular classroom setting, where they benefit from and enjoy a regular school system that meets their learning needs [24]. The research reports have showed that inclusive education could be effective for all learners thanks to the social, emotional and educational benefits of activities that involve students with different needs, preferences and personal resources [25]. These goals have been served by the introduction of solutions based on universal design learning (UDL), rational adjustments of curricula and lesson scenarios and selection and application of appropriate textbooks and training materials for the teaching–learning process [26,27]. If the implemented principles of UDL do not remove the barriers that prevent some students from accessing the learning process, it is necessary to introduce rational adjustments and modifications in the following areas: equipment, lighting, timetables, methods, forms, teaching content, didactic aids and methods of school assessment. The sudden switch to ERL due to the pandemic and crisis management made the application of inclusive education very difficult. 

The undertaken study aims to answer two research questions:

Q1: What are the predominant approaches to supporting children’s ERL that are used by parents of SEN students?

Q2: What are the predictors of the predominant approaches to supporting children’s ERL that are used by parents of SEN students?

## 2. Methodology of Research

### 2.1. Sample of Research

In total, 421 parents (393 females, 28 males; see [28,29] for justification of the predominance of women over men in Social Science studies) of primary school students participated in the study; 83 people (20%) were parents of SEN children. The parents represented students at all stages of primary education: (a) grades 1–3, with 147 students in total, including 15 with SEN (10%); (b) grades 4–6, with 175 students in total, including 42 with SEN (24%); (c) grades 7–8, with 99 students in total, including 26 with SEN (26%). Students represented 45 different schools. Participation in the study was voluntary, and the respondents did not receive any remuneration. They were recruited through social media, groups of teachers, and parents of primary school students. The procedure was approved by the local ethics committee. The study was conducted in the early stage of the COVID-19 pandemic and restrictions, in April and May 2020. Due to the epidemiological situation, in Poland, ERL was introduced on 16 March 2020. Most classes in schools at the time were conducted in an asynchronous mode.

### 2.2. Instruments and Procedures

The parents who participated in the study completed an electronic 66-item questionnaire. It consisted of three parts, each of which corresponded to a key area of remote learning: (1) ways to support the child in their ERL; (2) perceived barriers to the implementation of ERL; (3) perceived benefits of ERL. The statements were rated on a Likert scale (ranging from 0 = “strongly disagree” to 5 = “strongly agree”). Factor analyses carried out separately for individual parts of the questionnaire allowed distinguishing of specific subscales (Table 1).

### 2.3. Data Analysis

The analysis of the results with IBM SPSS Statistics 26 was carried out in three stages. Firstly, a two-step cluster analysis allowed parental strategies for supporting children in ERL to be distinguished. The analyses were conducted for all parents, as their goal was to identify types of parental involvement and conduct further comparisons between groups. In the second stage, a Z test for two independent proportions was applied to compare the parents of children with and without SEN in terms of identifying the dominant approaches. The third stage applied a stepwise regression and focused on establishing the predictors of the SEN parents’ strategies of involvement in ERL.

## 3. Results of Research

**Figure 1:**
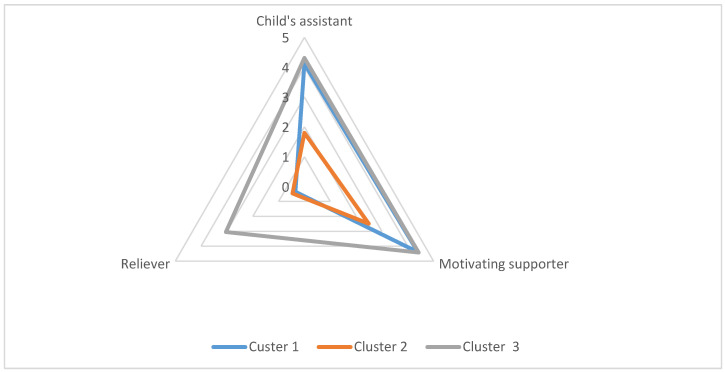
The three types of parental engagement in children’s ERL (Child’s assistant, Motivating supporter and Reliever) in the three distinguished clusters.

1. Three distinguishable clusters that indicate parents’ approaches to their involvement in their children’s ERL were observed (see Figure 1 for polar plots). Groups did not differ significantly in terms of age, gender, number of children, or other demographic variables. The collected data have been presented in Table 2.

**Cluster 1: Committed Teacher** (**CT**). This included parents who were extensively involved in the ERL of their children in a substantive and logistic way that motivates them to work, but these parents did not perform educational tasks for their children (219 parents). They spent on average approximately 3 h and 23 min a day supporting their children in ERL (M = 3.35; SD = 1.71).

**Cluster 2: Autonomy-Supporting Coach** (**ASC**). This included parents who were less likely to assist their children in ERL and more often supported them emotionally and motivated them to complete school tasks (106 parents). Such parents rarely relieved the child. They emphasized the importance of the child’s independence in the learning process, they devoted the least time to the ERL of their children, with only about 2 h a day (M = 1.99; SD = 1.89).

**Cluster 3: Committed Teacher and Reliever** (**CTR**). This included parents who extensively assisted and motivated the children but openly declared that they performed some school tasks for them (96 parents). This group of parents spent about 4 h and 20 min a day on the ERL of their children (M = 4.29; SD = 2.34).

2. The comparisons have showed that parents of SEN students (40%) used the CT strategy significantly less often (Z = 2.37; *p* < 0.01) than parents of non-SEN students (55%). A reversed pattern of the results was observed in the case of the CTR strategy, applied significantly more often by parents of SEN students (38%) than parents of non-SEN students (19%; Z = 3.67; *p* < 0.001). There was no difference between SEN and non-SEN parents in the frequency of using the ASC strategy.

More detailed analyses in the group of parents of SEN students revealed two dominant strategies for engaging in children’s ERL: CT strategy (applied by 40% of the sample) and CTR strategy (applied by 38% of the sample). Only about 20% of the parents of SEN students used the ASC strategy; this strategy was significantly less frequent compared to the other two strategies in this group of parents (for CT Z = 2.35; *p* < 0.01; for CTR Z = 2.19; *p* = 0.01).

Parents of SEN and non-SEN students also differed in the amount of time devoted to the remote education of their children. In general, the former devoted significantly more time to their children’s ERL (M rank = 257.38) than the parents of non-SEN students (M rank = 199.61; U = 10177.50; Z = −3.93; *p* < 0.001). Differences in this respect were also identified between parents of SEN students representing the three indicated clusters (Kruskal-Wallis test, *p* < 0.001).

3. The strongest predictor of application of the CT strategy by parents of SEN students was excessive demands on the school and teachers, and the model that took this predictor into account proved to be well-suited to the collected data (F (1.81) = 19.93; *p* < 0.001). The observed strength and direction of the impact of this predictor was moderate and positive (β = 0.44; t = 4.46; *p* < 0.001). This might suggest that the more the parents of SEN students perceived the requirements of the school as excessive and chaotic, the more they became involved in assisting their children in ERL. The inadequacy of the requirements related to lack of clear rules and demands but also overloading the child with too many tasks, content too difficult for independent learning and the child’s fatigue due to too much work explained approximately 20% of the CT strategy variance (*R^2^* = 0.197).

Further comparisons showed that the strongest predictor of CTR was the child’s motivation to study (F (1.81) = 28.03; *p* < 0.001). A lack of motivation to study perceived by the parents of SEN students significantly contributed to engaging in ERL by completing school tasks for the child (β = 0.51; t = 5.29; *p* < 0.001). It explained almost 26% of the variability in the results regarding the willingness to perform school tasks for the child (*R^2^* = 0.257).

## 4. Discussion

The sample of the parents participating in the study was dominated by the CT strategy, which was related to the high involvement in the children’s ERL through explaining new or difficult content, assisting them in finding solutions and motivating them to study. An important element of this strategy was also involvement as a technical assistant, which means helping with the organization and preparation of materials, following the school calendar or contact with teachers and tutors if needed. This strategy was relatively less common in the population of parents of SEN students. On the other hand, parents of SEN students were relatively more likely than other parents in the sample to apply the CTR strategy, which involves performing school tasks for a child.

Parents of SEN students were significantly more involved than parents of non-SEN students in ERL: they devoted more time to the education of their children and more often helped them do their school tasks. This seems natural due to SEN students’ less independence and greater need for support. However, Szumski and Karwowski [3] associated this type of parental support with mechanical involvement and pointed to its negative effects on the knowledge, skills and independence of SEN students in the long term.

The performed regression analysis allowed the reasons for this mechanical involvement of parents of SEN students to be explained. The main explanatory variable in the undertaking the PI strategy could be excessive demand and chaos (CT), whilst carrying out educational tasks for the child (CTR) could be explained by their lack of motivation to study. It is worth adding that shaping the motivation of SEN students to study was one of the main areas developed during individual therapeutic classes, which were suspended during ERL [15]. Maladjusted school requirements, unsuitable educational activities and individual problems resulting from children’s deficits could reduce their motivation to ERL.

The obtained data indicated that in the ERL in the pandemic chaos, in which there was a lack of regular therapeutic classes, SEN-parents tried to protect their children from the frustration associated with the excessive burden caused by inadequate school requirements and subsequent failures by relieving or accelerating the learning process by focusing on visible effects (e.g., doing homework) and ignoring the natural phases of knowledge formation and the methodology of problem solving. Such parental strategy could be related to the lack of adaptation of materials and tasks to the educational needs of this group of children [30].

The inadequacy of teachers’ requirements perceived by SEN-parents, together with a perceived lack of motivation of SEN students to study, seemed to explain the parental tendency to perform school tasks for such children. Moreover, unclear communication, which is the key barrier to the development of remote education [31], could have exacerbated these difficulties. Therefore, ERL did not meet the assumptions of inclusive education [32], leading SEN-parents to the engagement exceeding their qualifications.

The ASC strategy was the least popular way of parents getting involved in their children’s ERL. Parents using this strategy tried to motivate their children to study, support them emotionally and strengthen their independence. This type of commitment was an aspect of long-term support which was not focused on instant effects but consisted in investing in competences that will be later useful in life [4]. Despite being the rarest approach, the ASC strategy could be seen as especially important for SEN students in developing their independence and self-efficacy as a priority in education [33]. Moreover, this form of support was an aspect of subtle PI, which, as shown in the meta-analysis by Yong Tan, Ch., Lyu, M. and Peng [5], was strongly correlated with students’ achievements due to the development of their general positive attitude towards learning. In such circumstances, parental encouragement and reinforcement can provide healthy scaffolding for a child. However, parents’ autonomy-promoting attitudes (ASC) might conflict with the requirements of teachers, who were focused mainly on assessing homework and knowledge during ERL. Such teachers’ attitudes did not create an opportunity for students to develop general competences that could be important in life. The differences between parents’ and teachers’ goals could be the key barriers to the efficient PI [34]. The rarity of using the autonomy-supporting strategy in comparison to the popularity of the strategy that involved relieving a child in their school tasks might, therefore, indicate that parents matched the expectations of teachers [35], who were unfortunately obliged to gather evidence of the full implementation of the core curriculum during ERL.

To sum up, the way of supporting children during ERL might be crucial in the process of gaining new knowledge and skills by them. Obtained results indicate that SEN- parents applied different strategies, among which the one supporting students’ autonomy appeared to be the rarest and the most beneficial at the same time. The ASC strategy. as the most constructive, could create an opportunity for students to develop their independence and responsibility for school achievements. Greater independence can be also associated with students’ greater intrinsic motivation [14]. It might have a huge impact on the effectiveness of the learning process [27]. Such an approach could be promoted by reducing chaos and exaggerated demands in every case of instability and disruption.

The raised issues related to the causes and consequences of differential PI strategies in children’s remote education require further research and analysis. It would be interesting to verify whether, in post-pandemic or standard learning and teaching conditions, the demonstrated differences in parental involvement of SEN and non-SEN students are equally pronounced. It would be interesting to see whether the synchronicity of the remote classes or the lack of it (as was the case in the first stage of the pandemic) matters for the extent and dominance of PI strategies and the range of activities undertaken by parents.

## Figures and Tables

**Table 1:** The subscales of the questionnaire.

Module	Subscale	Number of Items	Cronbach’s Alpha
*Parental ways to support children ERL*	Assistance (sending work files, printing materials, controlling the course of activities, installing equipment, etc.)	5	0.799
	Motivating support (translating difficult topics, motivating to learn, rewarding, etc.)	5	0.821
	Relieving (completing the tasks for the child)	1	0.761 (factor load)
*Perceived barriers of ERL*	Inadequate requirements (child overload and fatigue)	10	0.94
	Parent’s adaptive stress (difficulties in adapting and combining the roles of parent, teacher and employee)	8	0.849
	Methodical and communication chaos (lack of clear guidelines and rules)	6	0.893
	Lack of child’s motivation (child’s reluctance and boredom)	5	0.844
	Limited availability of ERL (lack of access to materials and equipment)	3	0.693
	Limited social relations (no contact with teachers and colleagues)	2	0.794
*Perceived benefits of ERL*	Child development (new skills, increasing independence)	5	0.815
	Child comfort (avoiding disliked peers, no heavy backpack, freedom of action)	3	0.543
	Educational attractions (access to various sources and attractive educational content)	2	0.505

**Table 2:** Parents’ involvement in the ERL of their children: comparison of clusters.

Factors Distinguished in the Study Regarding Parental Involvement in a Child’s ERL	Cluster			Difference		Post Hoc
	1 CT *n* = 219 (52%)	2 ASC *n* = 106 (25%)	3 CTR *n* = 96 (23%)	F	*p*	*p* < 0.001
**Child’s assistant**	4.12	1.8	4.32	303.29	*p* < 0.001	2 < 1, 3
**Motivating supporter**	4.4	2.48	4.42	309.55	*p* < 0.001	2 < 1, 3
**Reliever**	0.35	0.46	3.04	455.12	*p* < 0.001	3 > 1, 2

## Data Availability

The data presented in this study are available on request from the corresponding author.
